# Association of ambient Particulate matter 2.5 with intensive care unit admission due to pneumonia: a distributed lag non-linear model

**DOI:** 10.1038/s41598-017-08984-x

**Published:** 2017-08-17

**Authors:** Zhongheng Zhang, Yucai Hong, Ning Liu

**Affiliations:** Department of emergency medicine, Sir Run-Run Shaw Hospital, Zhejiang University School of Medicine, Hangzhou, 310016 China

## Abstract

Air pollution in China has become a major environmental problem. There is a lack of evidence on the impact of haze (especially PM2.5) on intensive care unit admission due to pneumonia (ICUp). We hypothesized that PM2.5 was independently associated with ICUp and there was a non-linear time lag effect. All ICU admissions occurred from January 1, 2014 to December 31, 2016 in Sir Run-Run Shaw hospital were included in the study. The primary reasons for admission were categorized into pneumonia and non-pneumonia. Distributed lag non-linear model (DLNM) was built to account for the effect of air quality parameters in both value and temporal lag dimensions. There was a total of 7487 ICU admissions during the study period, including 391 admissions due to pneumonia. The DLNM showed that the relative risk (RR) of ICUp increased with PM2.5 concentrations. At a PM2.5 concentration of 200 mcg/m3, the RR increased from 1.06 (95% CI: 0.57–1.95) at day 0 to 1.40 (95% CI: 1.05–1.86) at day 3, and returned normal at day 6 (RR: 1.13; 95% CI: 0.83–1.55). The study showed that PM2.5 was independently associated with the risk of ICUp, and the maximum effect occurred at 3 to 4 days after exposure.

## Introduction

With rapid industrialization and economic development, China has been plagued with air pollution in recent years. Many large cities and urban areas have been frequently attacked by haze episodes, and the problem is much worse in northern areas and in cold seasons^1^. The most important component of haze is Particulate matter 2.5 (PM2.5), which is thought to be associated with combustion of fossil fuels, industrial processes, agriculture activities, construction dust, and household space heating^2, 3^. It is reported that fewer than 1% of the 500 largest cities in China meet air quality standards for PM2.5 of an annual mean of less than 10 mcg/m3, as recommended by the world health organization (WHO)^4^.

In medical literature, haze with PM2.5 has been identified as an important trigger of asthma and exacerbation of chronic obstructive airway disease (COPD)^5^. Short or long-term exposure to PM2.5 is associated with a series of respiratory symptoms such as cough, wheezing and dyspnea^6, 7^. Furthermore, epidemiological studies showed that PM2.5 concentration was positively associated with mortality due to a number of causes^8^. There have been evidence that hospitalization with pneumonia is associated with haze episode^9, 10^, and the pathogenesis may be mediated via PM2.5-associated metals^11^. However, most of these studies are based on data from outpatient visits or death registration, and there is no report on whether haze episode can cause critical illness, leading to intensive care unit (ICU) admission due to respiratory reasons. The study aimed to investigate the association between ICU admissions due to pneumonia and haze episode. We hypothesized that PM2.5 was associated with increased risk of ICU admission due to pneumonia (ICUp).

## Materials and Methods

### Study population

All ICU admissions occurred from January 1, 2014 to December 31, 2016 in Sir Run-Run Shaw hospital were included in the study. Data were extracted from electronic health records system for ICU patients. The primary reasons of ICU admission were recorded for each admission, which were categorized into pneumonia and non-pneumonia. Because the primary goal of the study was to investigate the association between air pollution and ICUp, patients with other diagnosis were classified as non-pneumonia patients. Patients admitted to ICU without pneumonia were used as comparators. Data on demographics such as age and gender were collected. Severity scores such as Acute Physiology and Chronic Health Evaluation II (APACHEII) and Mortality Prediction Model II (MPMII) scores were obtained from the database. Admission sources included emergency room, emergency surgery, ICU, outpatient, postoperation and ward. Although there are differences between indoor and outdoor air qualities, there is evidence that outdoor pollution can greatly affect indoor environment^12, 13^. Thus, patients transmitted to ICU from all places were included in our analysis. Discharge outcomes included death, against-advice discharge (AAD), transferring to ward and other hospitals. The length of stay in ICU was also calculated based on the date of admission and discharge. The study was approved by the ethics committee of Sir Run-Run Shaw hospital (20170113–1), and informed consent was waived due to retrospective design of the study.

### Air quality parameters

Air quality parameters included PM2.5, PM10, sulfur dioxide (SO_2_), carbon monoxide (CO), nitrogen dioxide (NO_2_) and ozone (O_3_). There were 11 monitoring sites in Hangzhou city. Because patients admitted to our hospital roughly covers the entire Hangzhou city, the average value of the 11 sites was employed as the exposure levels. All air quality parameters were recorded on a daily basis. Historical and real time air quality data could be obtained from the national air quality study website (https://www.aqistudy.cn/). Because ICUp could be affected by seasonal temperature, we incorporated daily mean temperature to adjust for the confounding. Seasonal variation in ICUp was also accounted for in the study by tagging date with one of the four seasons. According to the Chinese lunar calendar, the winter, spring, summer and fall begin on November 7^th^, February 4^th^, May 5^th^ and August 7^th^, respectively.

### Statistical analysis

Continuous variables were expressed as mean (standard error) and compared using parametric student t test. Categorical variables were expressed as the number and proportions, and compared using Chi-square test. Clinical characteristics between ICUp and non-pneumonia causes of ICU admission (ICUnonp) were compared. Furthermore, air quality parameters on the day of ICU admission between ICUp and ICUnonp were compared^14^.

Multivariable logistic regression model was employed to explore independent air quality predictors of ICUp^15^. Variables included all air quality parameters, daily temperature and day of week. There were variations in the volume of ICU admission between working days and weekends, thus the seven week days were included in the model as dummy variables. To explore the time-lag effect of haze exposure on ICUp, we assumed a 4-day or 7-day lag between haze exposure and ICU admission. In other words, the logistic regression model was fit by incorporating air quality 4 days or 7 days prior to the ICU admission.

We assumed that the dependency of ICUp on air quality had delayed effects. Thus, an additional dimension, temporal dependency of exposure and outcome, was required to characterize the model. Distributed lag non-linear model (DLNM) is such a model that the relationship between air quality parameters and ICUp is described both along the usual pace of predictor and in additional dimension of temporal lags^16^. Temperature, PM2.5 and day of week were included in the DLNM, because they were found to be independently associated with ICUp. Polynomials functions with 2-degrees of freedom were assigned to the variable PM2.5 in both value and lag spaces. Natural cubic B-spline functions were given to the temperature variable. A lag effect at a maximum of 7 days were allowed in the DLNM. The day of week was modelled without lag effect. The outcome of the DLNM was the count of daily ICUp, and Poisson model was used. All statistical analyses were performed using R (R version 3.2.3). Two-tailed p < 0.05 was considered statistically significant.

## Results

**Figure 1:**
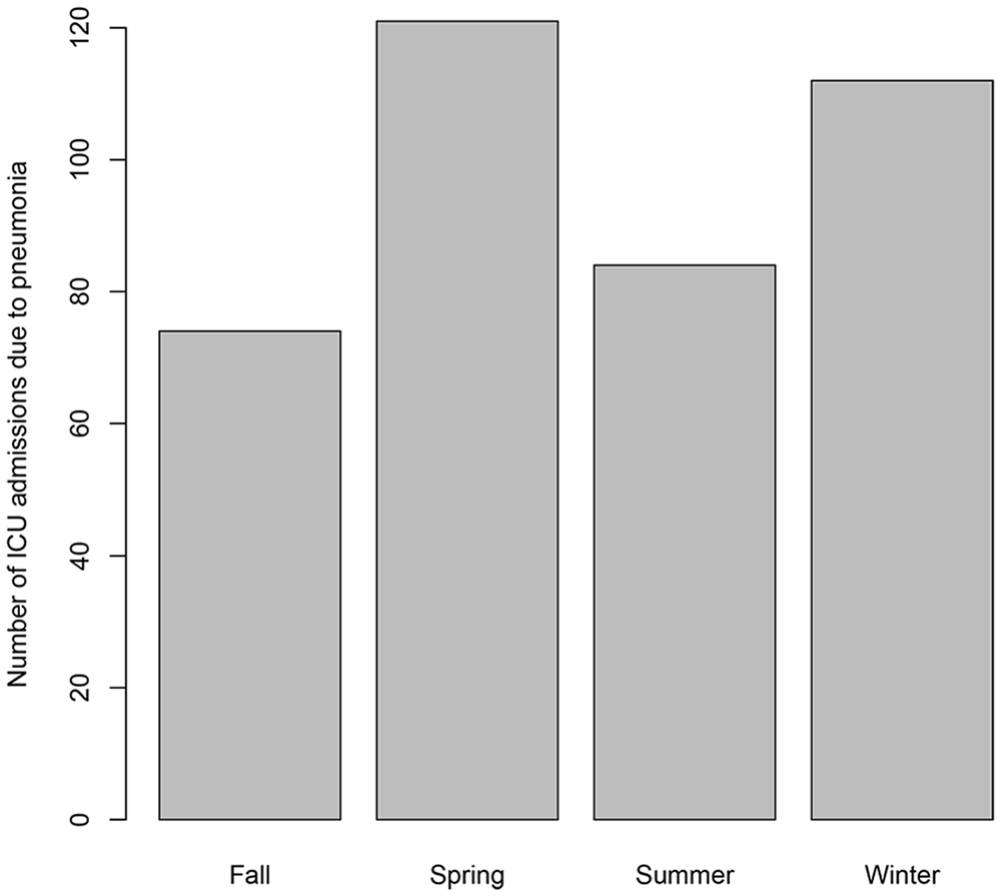
Seasonal variation of ICU admissions due to pneumonia (ICUp). There were more number of ICUp in winter and spring than that in fall and summer (*χ* ^2^ = 15.3, df = 3, p = 0.002).

From January 1, 2014 to December 31, 2016, there was a total of 7487 ICU admissions occurred in our hospital. Three hundred and ninety-one of them were admitted with the primary diagnosis of pneumonia (Table 1). Patients with pneumonia were significantly older (68.2 ± 15.4 vs. 60.3 ± 16.2 years; p < 0.001), had higher MPMII (29.6 ± 19.4 vs. 21.4 ± 18.9; p < 0.001) and APACHEII scores (21.4 ± 9.3 vs. 15 ± 8.6; p < 0.001). Pneumonia patients were more likely to be admitted from emergency room. Pneumonia patients showed higher risk of death (8.7% vs. 2.3%; p < 0.01) and AAD (20.7% vs. 7.9%; p < 0.01). It was colder on the day of admission for ICUp than ICUnonp (16.3 ± 9.1 vs. 18.4 ± 8.6 °C; p < 0.001). Air pollutants such as PM2.5 (61.6 ± 33.7 vs. 54.8 ± 29.2 mcg/m3; p < 0.001), PM10 (93 ± 47.6 vs. 85.8 ± 42.7 mcg/m3; p = 0.003), and SO2 (17 ± 9 vs. 15.9 ± 8.6 mcg/m3; p = 0.028) were significantly higher on the day of admission for ICUp than ICUnonp. Seasonal variation of ICUp is shown in Fig. 1. It appears that the number of ICUp was higher in winter and spring, than that in summer and fall (*χ* ^2^ = 15.3, df = 3, p = 0.002). The number of ICUp was the lowest in fall, which was thus used as the reference level in subsequent multivariable analysis.

**Table 1:** Clinical characteristics of included patients and air quality conditions on the day of admission.

	Overall (n = 7487)	Non-pneumonia (n = 7096)	Pneumonia (n = 391)	p
Age (mean, sd)	60.7 (16.3)	60.3 (16.2)	68.2 (15.4)	<0.001
Gender (M, proportion)	4643 (0.620)	4375 (0.617)	268 (0.685)	0.007
MPMII	21.9 (19.1)	21.4 (18.9)	29.6 (19.4)	<0.001
APACHEII	15.4 (8.8)	15.0 (8.6)	21.4 (9.3)	<0.001
**Admission sources (No. proportion)**				
Emergency	2923 (0.390)	2665 (0.376)	258 (0.660)	<0.01
Emergency surgery	653 (0.087)	653 (0.092)	0	<0.01
ICU	126 (0.017)	110 (0.016)	16 (0.041)	<0.01
Outpatient	69 (0.009)	67 (0.009)	2 (0.005)	<0.01
Postoperation	2516 (0.336)	2515 (0.354)	1 (0.003)	<0.01
Ward	1200 (0.160)	1086 (0.153)	114 (0.292)	<0.01
**Discharge (No. proportion)**				
AAD	643 (0.086)	562 (0.079)	81 (0.207)	<0.01
Die	194 (0.026)	160 (0.023)	34 (0.087)	<0.01
Discharge	169 (0.023)	155 (0.022)	14 (0.036)	<0.01
To other hospital	221 (0.030)	179 (0.025)	42 (0.107)	<0.01
To ward	6216 (0.830)	5999 (0.845)	217 (0.555)	<0.01
Length of stay (hours)	160.0 (500.1)	141.2 (444.1)	503.2 (1044.1)	<0.001
**Air quality on the day of ICU admission**				
PM2.5 (mcg/m3)	55.2 (29.5)	54.8 (29.2)	61.6 (33.7)	<0.001
PM10 (mcg/m3)	86.1 (43.0)	85.8 (42.7)	93.0 (47.6)	0.003
SO_2_ (mcg/m3)	16.0 (8.6)	15.9 (8.6)	17.0 (9.0)	0.028
CO (mg/m3)	0.9 (0.2)	0.9 (0.2)	0.9 (0.3)	0.002
NO_2_ (mcg/m3)	46.3 (16.6)	46.2 (16.4)	47.5 (18.4)	0.185
O_3_ (mcg/m3)	111.3 (57.6)	111.7 (57.8)	104.1 (54.4)	0.007
Temperature (°C)	18.3 (8.6)	18.4 (8.6)	16.3 (9.1)	<0.001

Abbreviations: MPMII: Mortality Prediction Model II; APACHEII: Acute Physiology and Chronic Health Evaluation II; AAD: against advice discharge; ICU: intensive care unit; PM: particulate matter; SO_2_: sulfur dioxide; CO: carbon monoxide; NO_2_: nitrogen dioxide; O_3_: ozone.

In multivariable regression model, temperature (OR: 0.97; 95% CI: 0.95–1.00; p = 0.031) and PM2.5 (OR: 1.01; 95% CI: 1.00–1.02; p = 0.021) on the day of ICU admission were independently associated with ICUp. The effect of PM2.5 persisted at the 4^th^ day (OR: 1.02; 95% CI: 1.00–1.03; p = 0.005), and disappeared on the 7^th^ day (OR: 1.00; 95% CI: 0.99–1.02; p = 0.478). As expected, days of weekend (e.g. Saturday and Sunday) were consistently associated with increased risk of ICUp (Table 2). As compared with fall, none of the other seasons were significantly associated the risk of ICUp, indicated that the seasonal variation in ICUp was attributable to the temperature and air qualities.

**Table 2:** multivariable regression model investigating independent predictors of ICU admission due to pneumonia.

	Air quality on the day of ICU entry				Air quality 4 days before ICU entry				Air quality 7 days before ICU entry			
Variables	OR	LCI	UCI	P	OR	LCI	UCI	P	OR	LCI	UCI	P
Temperature	0.97	0.95	1.00	0.031	0.98	0.96	1.01	0.133	0.97	0.95	0.99	0.010
PM2.5	1.01	1.00	1.02	0.021	1.02	1.00	1.03	0.005	1.00	0.99	1.02	0.478
PM10	0.99	0.99	1.00	0.159	0.99	0.99	1.00	0.111	1.00	0.99	1.00	0.536
SO_2_	0.99	0.98	1.01	0.287	0.98	0.97	1.00	0.061	0.99	0.98	1.01	0.397
CO	1.10	0.51	2.34	0.803	0.76	0.35	1.61	0.479	1.40	0.66	2.95	0.375
O_3_	1.00	1.00	1.00	0.833	1.00	1.00	1.00	0.346	1.00	1.00	1.00	0.827
**Day of week (Monday as reference)**												
Tuesday	1.28	0.86	1.94	0.229	1.26	0.84	1.90	0.270	1.26	0.84	1.90	0.273
Wednesday	1.44	0.97	2.15	0.071	1.39	0.94	2.08	0.104	1.40	0.94	2.09	0.098
Thursday	1.47	0.99	2.20	0.057	1.38	0.93	2.08	0.113	1.42	0.96	2.14	0.085
Friday	1.53	1.03	2.29	0.038	1.53	1.02	2.29	0.039	1.57	1.05	2.35	0.028
Saturday	2.55	1.72	3.83	< 0.001	2.59	1.74	3.88	< 0.001	2.59	1.74	3.88	0.000
Sunday	2.12	1.39	3.25	0.001	2.15	1.41	3.30	< 0.001	2.08	1.36	3.20	0.001
**Seasons (Fall as reference)**												
Spring	1.26	0.86	1.85	0.244	1.36	0.90	2.03	0.141	1.13	0.74	1.72	0.574
Summer	1.09	0.78	1.52	0.626	0.99	0.71	1.38	0.968	1.05	0.75	1.45	0.786
Winter	0.95	0.59	1.54	0.849	1.26	0.78	2.04	0.349	0.89	0.55	1.45	0.645

Abbreviations: PM: particulate matter; SO_2_: sulfur dioxide; CO: carbon monoxide; NO_2_: nitrogen dioxide; O_3_: ozone; LCI: lower limit of 95% confidence interval; UCI: lower upper of 95% confidence interval; OR: odds ratio.

**Figure 2:**
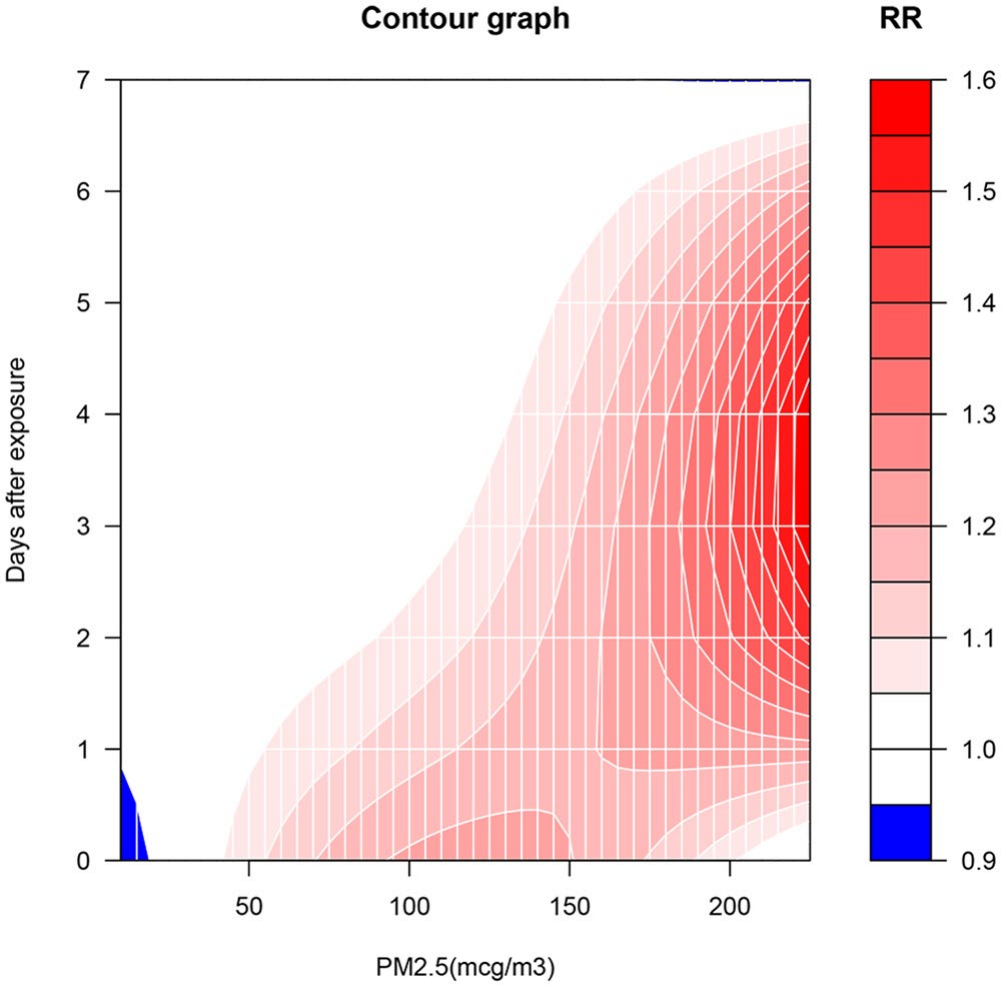
Contour plot showing adjusted effect of PM2.5 on relative risk (RR) of ICUp, setting the reference at PM2.5 of 30 mcg/m3. At a PM2.5 < 50 mcg/m3, there was no additional risk of ICUp. The RR increases with increasing PM2.5 concentrations. Furthermore, the effect size of PM2.5 reached its plateau at 3 to 4 days after exposure. Thereafter, the effect decreased gradually and returned to normal after 6 to 7 days.

**Figure 3:**
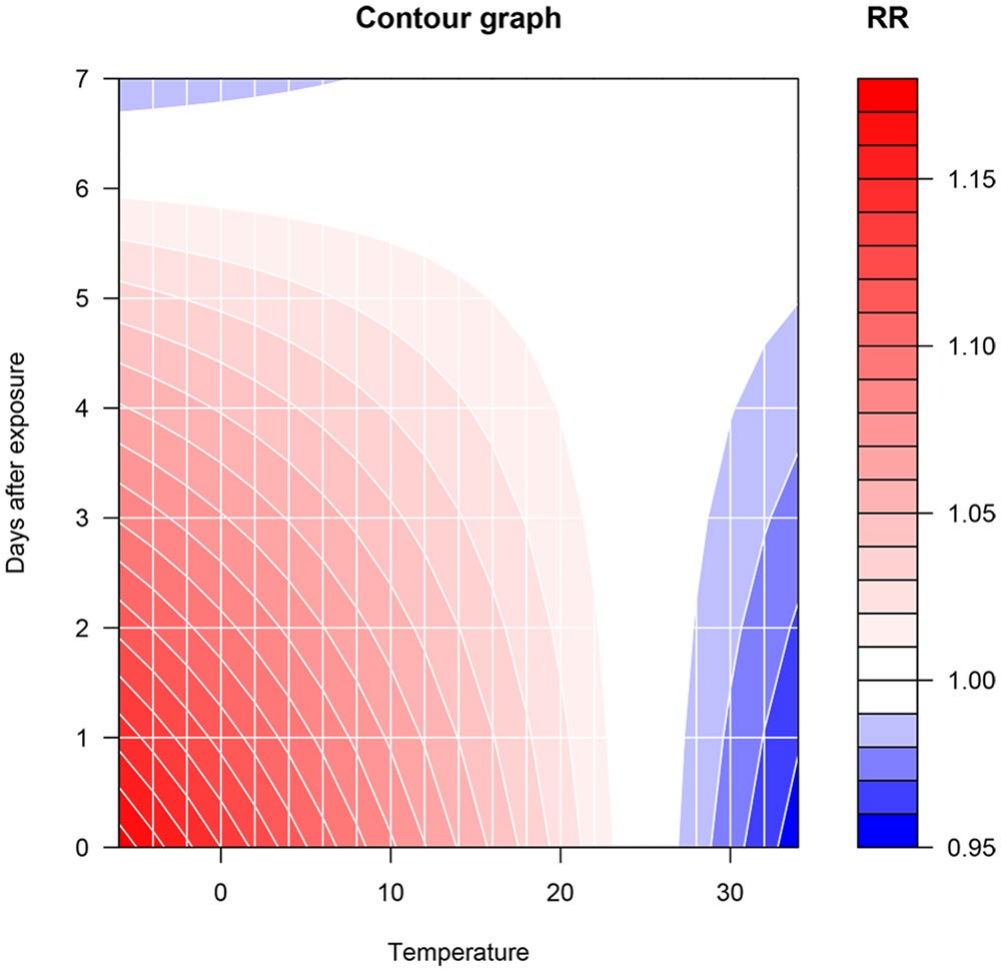
The adjusted effect of temperature on ICUp. As expectedly, low temperature was associated with increased risk of ICUp. In contrast to the effect of PM2.5, temperature appeared to have immediate effect after exposure.

The results of DLNM are shown in Table 3. It appeared that the relative risk (RR) of ICUp increased with PM2.5 concentrations. At a PM2.5 concentration of 200 mcg/m3, the RR increased from 1.06 (95% CI: 0.57–1.95) at day 0 to 1.40 (95% CI: 1.05–1.86) at day 3, and returned normal at day 6 (RR: 1.13; 95% CI: 0.83–1.55). Figure 2 is a contour plot showing adjusted effect of PM2.5 on ICUp. At a PM2.5 < 50 mcg/m3, there was no additional risk of ICUp. The RR increased with increasing PM2.5 concentrations. Furthermore, the effect of PM2.5 reached its plateau at 3 to 4 days after exposure. Thereafter, the effect decreased gradually and returned to normal after 6 to 7 days. Figure 3 shows the effect of temperature on ICUp. As expectedly, low temperature was associated with increased risk of ICUp. In contrast to the effect of PM2.5, temperature appeared to have immediate effect after exposure.

**Table 3:** Relative risks of ICU admission due to pneumonia at specific combinations of PM2.5 values and lag times.

PM2.5 (mcg/m3)	0 day	1-day lag	2-day lag	3-day lag	4-day lag	5-day lag	6-day lag	7-day lag
10	0.91 (0.81,1.02)	0.96 (0.90,1.02)	1.00 (0.94,1.06)	1.03 (0.96,1.09)	1.04 (0.98,1.11)	1.04 (0.99,1.10)	1.03 (0.97,1.10)	1.01 (0.90,1.14)
50	1.08 (0.99,1.18)	1.04 (0.99,1.09)	1.01 (0.97,1.06)	0.99 (0.94,1.04)	0.98 (0.93,1.02)	0.97 (0.93,1.01)	0.98 (0.93,1.02)	0.99 (0.91,1.08)
100	1.21 (0.98,1.49)	1.13 (1.00,1.27)	1.07 (0.96,1.18)	1.02 (0.91,1.14)	0.99 (0.88,1.10)	0.97 (0.88,1.06)	0.96 (0.86,1.08)	0.97 (0.78,1.20)
150	1.20 (0.88,1.65)	1.19 (1.00,1.43)	1.17 (1.02,1.35)	1.14 (0.98,1.34)	1.11 (0.95,1.29)	1.06 (0.93,1.21)	1.01 (0.86,1.18)	0.95 (0.71,1.29)
200	1.06 (0.57,1.95)	1.23 (0.86,1.74)	1.35 (1.03,1.76)	1.40 (1.05,1.86)	1.38 (1.04,1.82)	1.28 (1.00,1.65)	1.13 (0.83,1.55)	0.95 (0.54,1.66)

Note: distributed lag non-linear model was fit by incorporating PM2.5, mean temperature and week. PM2.5 value and its lag effect were modelled by polynomials function with 2 degrees of freedom. Temperature and its lag effect were modelled with nature cubic spline functions. A PM2.5 value of 30 mcg/m3 was employed as reference, and relative risks of other concentrations were compared to this reference.

## Discussion

The study for the first time reported that PM2.5 and temperature were independently associated with ICU admissions due to pneumonia (ICUp). The PM2.5 relative risk for ICUp increased immediately after exposure and reached a plateau at 3 to 4 days, thereafter the RR declined and returned to the baseline level 6 days after exposure. Low temperature was associated with increased risk of ICUp, especially when it dropped below 0 °C. Unlike the lagged effect for PM2.5, temperature appeared to have more immediate effect. Although other components of air quality showed significant associations with ICUp in univariate analysis, their effects disappeared after adjustment. This could be explained by correlations between PM2.5 and other pollutants.

PM2.5 is the most important contribution to air pollution in China, and the level is much higher than that reported in western countries. For example, the average PM2.5 level in Hangzhou was 50 mcg/m3, which was five times higher than that reported in the United States^17^. Therefore, health burden of air pollution can be prominent in mainland China. There is a large body of evidence showing that even a small increase (e.g. > 10 mcg/m3) in PM2.5 is associated with significant increases in hospital admissions and mortality due to cardiovascular and/or respiratory reasons^17–23^. However, all these studies were conducted in regions with daily PM2.5 concentrations between 10–30 mcg/m3 and there was lack of evidence on how high level PM2.5 could impact health. One study conducted in Jinan (e.g. a Chinese city with high annual PM2.5 level) found that elevated PM_2.5_ concentrations were associated with increased risk of pediatric hospital admissions for pneumonia^24^. Furthermore, previous studies primarily focused on outcomes such as outpatient visits or general ward admissions, which involved less severe forms of respiratory diseases^25, 26^. There is no data on the impact of high level PM2.5 on severe respiratory diseases requiring ICU admissions (e.g. critical illness). The study aimed to explore the impact of high level PM2.5 on ICUp. An interesting finding in the study was that there was a 3 to 4 days lag for PM2.5 exposure to take its maximum effect. This temporal lag probably reflected the time between disease onset and ICU admission. Sometimes, the ICU bed congestion may delay the admission^27^.

A strength of the study was the use of DLNM, allowing for modelling the relationship between air pollutants and a sequence of outcomes. The distribution of the delayed effect can be specified at different time points. In our model, we specified a maximum lag of 7 days. The DLNM also helped to account for the phenomenon of “harvest”. The so-called “harvest” is to describe a phenomenon that severe pneumonia may occur in fragile subjects immediately after exposure, leaving limited number of subjects at risk and the overall long-term impact is reduced^28^. Furthermore, we modelled non-linear effects in both lag and predictor dimensions. It could be reasonable that the effect size declined after a plateau and returned normal within a specific time period. Our data supported this hypothesis and the model appeared well fitted with natural cubic B-spline and polynomials functions^28, 29^.

Several limitations must be acknowledged in the study. This was a single center study that the result may not be well generalizable to other hospitals. There were many hospitals in Hangzhou and patients admitted to other hospitals were not included, leading to potential selection bias. However, our hospital is a university-affiliated tertiary care hospital and most patients with severe pneumonia requiring ICU admission were treated in the hospital. It was a good sampling of the overall population with severe pneumonia in Hangzhou city. Secondly, the average values of air qualities across 11 monitoring sites were used to represent the exposure. In fact, there were regional variations in air qualities, and thus patients from different regions had different exposure levels. In other words, the average exposure levels were not the true exposure levels for an individual patient. However, it was difficult to exactly determine the exposure level for individual patients, because patients were ambulatory and their living places usually did not match the one recorded in the database. Thirdly, humidity was not considered in the study due to unavailability of the data. It is probably that dry air may have negative impact on the health of respiratory tract.

In conclusion, the study showed that PM2.5 was independently associated with the risk of ICU admissions due to pneumonia (ICUp), and the maximum effect occurred at 3 to 4 days after exposure. Every effort should be made to protect susceptible individuals from haze exposure, especially when PM2.5 exceeds 150 mcg/m3.

### Ethics, consent and permissions

The study was approved by the ethics committee of Sir Run Run Shaw hospital. Informed consent was waived due to retrospective design of the study. The study was conducted according to Helsinki declaration.
